# Short-term wind speed prediction based on improved Hilbert–Huang transform method coupled with NAR dynamic neural network model

**DOI:** 10.1038/s41598-024-51252-y

**Published:** 2024-01-05

**Authors:** Jian Chen, Zhikai Guo, Luyao Zhang, Shanju Zhang

**Affiliations:** Water Conservancy College, North China University of Water Resources and Electric Power, Zhengzhou, 450046 China

**Keywords:** Atmospheric dynamics, Hydrology

## Abstract

Wind energy, as a renewable energy source, offers the advantage of clean and pollution-free power generation. Its abundant resources have positioned wind power as the fastest-growing and most widely adopted method of electricity generation. Wind speed stands as a key characteristic when studying wind energy resources. This study primarily focuses on predictive models for wind speed in wind energy generation. The intense intermittency, randomness, and uncontrollability of wind speeds in wind power generation present challenges, leading to high development costs and posing stability challenges to power systems. Consequently, scientifically forecasting wind speed variations becomes imperative to ensure the safety of wind power equipment, maintain grid integration of wind power, and ensure the secure and stable operation of power systems. This holds significant guiding value and significance for power production scheduling institutions. Due to the complexity of wind speed, scientifically predicting its fluctuations is crucial for ensuring the safety of wind power equipment, maintaining wind power integration systems, and ensuring the secure and stable operation of power systems. This research aims to enhance the accuracy and stability of wind speed prediction, thereby reducing the costs associated with wind power generation and promoting the sustainable development of renewable energy. This paper utilizes an improved Hilbert–Huang transform (HHT) using complementary ensemble empirical mode decomposition (CEEMD) to overcome issues in the traditional empirical mode decomposition (EMD) method, such as component mode mixing and white noise interference. Such an approach not only enhances the efficiency of wind speed data processing but also better accommodates strong stochastic and nonlinear characteristics. Furthermore, by employing mathematical analytical methods to compute weights for each component, a dynamic neural network model is constructed to optimize wind speed time series modeling, aiming for a more accurate prediction of wind speed fluctuations. Finally, the optimized HHT-NAR model is applied in wind speed forecasting within the Xinjiang region, demonstrating significant improvements in reducing root mean square errors and enhancing coefficient of determination. This model not only showcases theoretical innovation but also exhibits superior performance in practical applications, providing an effective predictive tool within the field of wind energy generation.

## Introduction

Wind energy, as a clean and environmentally friendly renewable energy source, provides a crucial solution to reducing reliance on fossil fuels and lowering greenhouse gas emissions^1^. According to data from the Global Wind Energy Council (GWEC), the global wind power capacity witnessed substantial growth in 2022, with a newly installed capacity of 77.6 GW, resulting in a total capacity of 906 GW, marking a 9% year-on-year increase^2^. Over the past five years, global wind power installation has shown a robust upward trend. The GWEC predicts a global increase of 680 GW in wind power capacity between 2023 and 2027, with 130 GW coming from offshore wind power. China’s onshore wind power capacity is expected to lead, reaching 300 GW, followed closely by Europe, nearing 100 GW. Consequently, the international community has increasingly focused on the development and utilization of wind energy in recent years^3^.

However, the rapid development of wind power faces constraints from various factors^4^. Typically, two crucial physical parameters defining wind power are wind speed and direction. Wind energy resource studies primarily revolve around these attributes, with wind speed being the primary metric^5^. For instance, grid networks demand stable and smooth inputs, yet natural wind, due to its stochastic intermittency, upon integration, can disrupt the grid, posing challenges to the scheduling and control of wind farms, along with potential safety concerns^6,7^. Therefore, accurate and effective wind speed prediction becomes paramount in addressing these issues. The establishment of an efficient short-term wind speed forecasting system is urgently needed^8^ to minimize harm to the grid and facilitate better unit adjustments and power control in wind farms.

Currently, scholars have researched and proposed various methods for wind speed forecasting to attain accurate predictions. These methods can be categorized into four groups: physical methods, statistical methods, artificial intelligence, and hybrid models^9,10^.

The physical methods for wind speed forecasting primarily rely on the Medium-Range Numerical Weather Prediction (NWP) models^11^. These methods involve obtaining meteorological elements such as wind speed, temperature, humidity, pressure, as well as information regarding topography, surface roughness, and wake effects at the location of the wind farm. These parameters are used to provide boundary conditions for the mathematical model to forecast wind speed and other meteorological elements. Therefore, physical methods can be viewed as a meteorological forecasting approach for wind speed, suitable for medium to long-term predictions, though short-term accuracy may not be as high. For instance, Theuer Frauke et al.^12^ discovered that forecast sensitivity to atmospheric conditions is lower than persistence-based forecasts using LIDAR. By assuming a stably calibrated logarithmic wind profile and extrapolating wind speed from measurements to forecast heights, a significant reduction in forecast error associated with height extrapolation was achieved. Cai et al.^13^ proposed a unified filtering framework for multi-range wind speed prediction, integrating short-term forecast models, Numerical Weather Prediction (NWP), and a smoothing term into a Bayesian filter-based unified framework, leading to improved short-term wind speed prediction performance. Louka, et al.^14^ applied the Kalman filtering algorithm to numerical wind speed forecasts. They simulated and predicted the future 24-h wind speeds of the Crete Island wind farm in Greece using wind speed data with resolutions of 12 km, 6 km, 1.5 km, and 0.5 km. The results showed that post-processing with Kalman filtering significantly improved prediction accuracy, eliminating any potential systematic errors.

Statistical forecasting models, based on a substantial amount of historical data, extract general trends and patterns in model variations. These models, also known as stochastic time series models, encompass multivariate linear regression models such as autoregressive (AR), moving average (MA), and autoregressive moving average (ARMA) models. However, linear regression models are more effective in fitting stationary sequences. They struggle to capture the intricate evolutionary characteristics of wind speed time series with strong nonlinear features, high stochasticity, and pronounced fluctuations, resulting in limited prediction accuracy. ARIMA models, on the other hand, leverage differencing operations, which possess robust capabilities in extracting deterministic information. They transform non-stationary sequences into differenced stationary sequences, and then fit them using ARMA models. The essence of the ARIMA model lies in the combination of differencing operations and ARMA models. For example, Jiang et al.^15^, considering the persistence of wind speed, initially employed an autoregressive moving average (ARMA) model to forecast wind speed sequences. The Boosting algorithm iterated the prediction results for T rounds, ultimately yielding the forecasted results. This approach was tested using real data from the eastern coast of Jiangsu Province, China, over the course of a year, and demonstrated a commendable level of prediction accuracy.

The artificial intelligence-based wind speed forecasting methods refer to the application of artificial intelligence algorithms in capturing the nonlinear features within historical wind speed data. Through data training, these methods ultimately achieve the prediction of wind speed time series characterized by nonlinear traits. Artificial intelligence approaches primarily encompass machine learning techniques and neural networks. For instance, Hazarika, et al.^16^ introduced the wavelet kernel-based least squares twin support vector regression (LSTSVR) model, which was applied in wind speed prediction with commendable outcomes. Deep learning, as a branch of artificial intelligence, involves networks with more neurons and hidden layers compared to shallow neural networks. Scholars have recently begun to apply deep learning techniques to wind speed prediction, including models like long short-term memory (LSTM) networks^17^ and convolutional neural networks (CNN)^18^. However, these models may exhibit drawbacks such as potential emergence of local minima, overfitting, and suboptimal performance on large-sample data.

The individual prediction models based on physics, statistics, or machine learning each have their own strengths and limitations. It is challenging to find a single model that consistently maintains optimal predictive performance across various scenarios. Extensive research has shown that the same prediction model may exhibit different performance levels in different regions or time periods. Therefore, selecting an appropriate prediction model for different forecasting environments has become a focal point in recent wind speed studies. Recognizing the limitations of individual models, many scholars have begun to explore different combinations and hybrid approaches. They combine single models mentioned earlier into advanced predictive models, utilizing different combination strategies to synergistically leverage the strengths of each model. This enhances the stability and applicability of the model's predictions in diverse scenarios^19^.

For instance, Chen et al.^20^ combined the autoencoder of Convolutional Neural Network (CNN) with Long Short-Term Memory Units (LSTM) to propose a novel deep learning model for two-dimensional regional wind speed prediction. Acikgoz Hakan et al.^21^ effectively preprocessed wind speed data using Variational Mode Decomposition (VMD) and employed a new deep neural network (WSFNet) for accurate multi-step ahead wind speed prediction. Liang et al.^22^ pretrained the Bi-LSTM model with historical data from four wind farms to identify wind speed feature parameters. This model was then utilized to predict wind speed for any wind farm. Pan et al.^23^ applied fast correlation filtering to rank and select the correlation attributes of wind speed sequences. They based their prediction on improved grey relational analysis of wind speed spatiotemporal correlations within turbine clusters. This resulted in the division of typical turbine multi-order neighborhoods and the reconstruction of wind speed information matrices for prediction. Jiang et al.^24^ assessed time series stationarity through time series charting and augmented Dickey-Fuller tests. They employed LSTM and GRU neural networks for wind speed prediction, yielding favorable predictive results. Wang et al.^25^ clustered wind turbine units using density peak algorithms, followed by applying long short-term memory network models for wind speed prediction within similar turbine clusters. Experimental results demonstrated the high predictive accuracy of this method.

In existing research, wind speed prediction mainly involves decomposing the historical measurement data of wind farms to establish predictive learning models. The prediction results of each component are directly superimposed, often overlooking the impact of IMF components with different frequencies on the prediction outcome. To address this, this paper proposes a mathematical analytical model weight optimization method to analyze the weight coefficients of each IMF component. Subsequently, an optimized HHT-NAR daily-level short-term wind speed prediction model is established. Firstly, the complementary ensemble empirical mode decomposition (CEEMD) method is introduced to decompose the original wind speed sequence. Then, the Hilbert transform (HT) method is employed to conduct spectral analysis on the obtained intrinsic mode functions (IMFs), taking advantage of its capabilities in handling nonlinearity and non-stationarity in signals. Different neural network models are established for prediction based on their respective spectral characteristics. Finally, the mathematical analytical model is utilized to determine the weight coefficients of each component. The final prediction result is obtained by weighted summation of the prediction results of each component. This methodology is applied to two different wind-rich areas in Xinjiang. Comparative analysis with other models is conducted to validate the reliability of the proposed model. The results indicate that the optimized model proposed in this paper has shown a significant improvement in reducing root mean square error and enhancing coefficient of determination. The results indicate that the optimized model proposed in this paper has shown a significant improvement in reducing root mean square error and enhancing coefficient of determination. This optimized model not only demonstrates theoretical innovation but also elevates the performance and generalizability of the model. It provides an effective predictive tool for the sustainable utilization of regional wind energy resources, potentially addressing the complex challenges faced by wind power generation.

## Wind speed prediction model based on an improved Hilbert–Huang transform

### An improved Hilbert–Huang transform method

The wind speed sequence data obtained in practice is often challenging to directly employ for prediction using neural network models due to potential inclusion of outliers and random noise^26^. Utilizing this data for prediction without preprocessing often results in significant errors. Therefore, it is imperative to employ data denoising techniques to enhance prediction accuracy. The Hilbert–Huang transform (HHT) is a processing method proposed by Huang et al. in 1998 for nonlinear, nonsmooth signals^27^, which is easy to implement, intuitive and efficient, with adaptivity, good time–frequency aggregation, completeness and reconfigurability^28^. The HHT theory consists of two parts, empirical mode decomposition (EMD) and Hilbert transform (HT). EMD method is one of the most commonly used techniques for data denoising. It decomposes complex time series data into several intrinsic mode functions (IMFs) and a trend component, enabling the application of Hilbert transform on each IMF. This allows for the derivation of the Hilbert spectrum for each component, ultimately facilitating the exploration and utilization of frequency domain characteristics of wind speed sequences. The method assumes that any complex signal consists of simple IMFs and each IMF component is independent of each other^29^. The specific procedure for decomposing the original wind speed time series is as follows.

1. Identify all the extreme and minimal points of the signal *V*(*t*) and fit the upper and lower envelopes of the original wind speed time series with the cubic spline function, respectively.
2. Calculate the mean value *m*_1_(*t*) of the upper and lower envelopes and subtract *m*_1_(*t*) from *V*(*t*) to obtain *h*_1_(*t*).
3. If *h*_1_(*t*) satisfies the IMF condition, note that *c*_1_(*t*) = *h*_1_(*t*), then *c*_1_(*t*) is the first IMF component and is the highest frequency component of the original wind speed time series; otherwise, treat *h*_1_(*t*) as a new *V*(*t*) and repeat the above steps *K* times until *h*_1_(*t*) satisfies the IMF condition.
4. Separating c_1_(t) from the original signal to obtain the residual component.
  1 $$ r_{1} \left( t \right) = v\left( t \right) - c_{1} \left( t \right) $$

Using *r*_1_(*t*) as the new original data, the above steps are repeated to obtain *n* IMF components and 1 residual component, and the results are as follows.

2 $$ \left\{ \begin{gathered} r_{1} \left( t \right) - c_{2} \left( t \right) = r_{2} \left( t \right) \hfill \\ r_{2} \left( t \right) - c_{3} \left( t \right) = r_{3} \left( t \right) \hfill \\ \vdots \hfill \\ r_{n - 1} \left( t \right) - c_{n} \left( t \right) = r_{n} \left( t \right) \hfill \\ \end{gathered} \right. $$

When *r*_*n*_(*t*) satisfies the given termination condition, the decomposition process ends, and the original wind speed time series can be expressed as:

3 $$ v\left( t \right) = \sum\limits_{i = 1}^{n} {c_{i} } \left( t \right) + r_{n} \left( t \right) $$

where *r*_*n*_ (*t*) is a residual function, representing the average trend of the signal; *c*_*i*_ (*t*) represents the components of signal at different time characteristic scales, and its scale increases successively from *c*_1_ (*t*) to *c*_*n*_ (*t*). The fractional termination condition Cosey convergence criterion proposed by Huang et al. is used, i.e., the standard deviation coefficient (SD) of the results of 2 consecutive IMF screening series is used as a criterion for judging, here denoted by S_d_, and defined as follows.

4 $$ S_{d} = \frac{{\sum\limits_{t = 0}^{T} {\left| {h_{i} \left( t \right) - h_{i - 1} \left( t \right)} \right|}^{2} }}{{\sum\limits_{t = 0}^{T} {\left| {h_{i - 1} \left( t \right)} \right|^{2} } }} \le \alpha $$

where *α* is a pre-set sufficiently small value, when *S*_*d*_ is less than or equal to *α*, the screening process is terminated; *T* is the number of original wind speed time series. For an arbitrary time series *X*(*t*), the Hilber transform is defined as the convolution *Y*(*t*) of *X*(*t*) with 1*/t*.

5 $$ Y\left( t \right) = \frac{1}{\pi }P\int_{ - \infty }^{ + \infty } {\frac{X\left( \tau \right)}{{t - \tau }}} d\tau $$

where *P* is the Kersey principal value, and the transformation holds for all *L*^*P*^ classes; *τ* is the integration variable; *t* is the current moment.

According to the above definition, the complex conjugate analytic signal *Z*(*t*) can be obtained from *X*(*t*) and *Y*(*t*).

6 $$ Z\left( t \right) = X\left( t \right) + jY\left( t \right) = a\left( t \right)e^{j\theta \left( t \right)} $$

Among them:

7 $$ \left\{ \begin{gathered} a\left( t \right) = \sqrt {X^{2} \left( t \right) + Y^{2} \left( t \right)} \hfill \\ \theta \left( t \right) = \arctan \left[ {Y\left( t \right)/X\left( t \right)} \right] \hfill \\ \end{gathered} \right. $$

At this point, the instantaneous frequency is as follows:

8 $$ f\left( t \right) = \frac{1}{2\pi }\frac{d\theta \left( t \right)}{{dt}} $$

However, when the components of a frequency band of the signal are discontinuous, or when there is intermittent noise interference, EMD may suffer from modal aliasing, which destroys the physical meaning implied by each IMF and reduces the decomposition accuracy^30^. In order to avoid the different modes of the signal in the decomposition process together, Complementary Ensemble Empirical Mode Decomposition (CEEMD) will be a pair of opposite numbers of positive and negative white noise as an auxiliary noise added to the source signal, in order to eliminate the original EMD decomposition of the reconstructed signal in the excess of the auxiliary white noise, and at the same time to reduce the decomposition of the number of iterations required to reduce the cost of computation^31^. CEEMD can be a very good solution to the phenomenon of mode mixing. The specific decomposition steps are as follows.

1. A pair of white noise with opposite sign and zero mean is randomly added to the original time series *Xt* to obtain two new series *M*_1_ and *M*_2_, one of which is denoted by $\omega_{t}$.
  9 $$ \left[ \begin{gathered} M_{1} \hfill \\ M_{2} \hfill \\ \end{gathered} \right] = \left[ {\begin{array}{*{20}c} 1 & 1 \\ 1 & { - 1} \\ \end{array} } \right]\left[ \begin{gathered} X_{t} \hfill \\ \omega_{t} \hfill \\ \end{gathered} \right] $$
2. The EMD algorithm is used to decompose *M*_1_ and *M*_2_ respectively to obtain two sets of IMF components and residual terms.
3. Repeat the above steps *N* times, *N* = 0*,* 1*,* 2*,…,* and the eigenmodal components of the CEEMD decomposition can be obtained by taking the mean value of the overall 2*N* modal components generated.
  10 $$ IMF_{j} = \frac{1}{2N}\sum\limits_{i = 1}^{2N} {IMF_{ij} } $$
  11 $$ R = \frac{1}{2N}\sum\limits_{i = 1}^{2N} {R_{i} } $$
  where *IMFj* denotes the *IMF* component of the decomposed sequence, 1 ≤ *j* ≤ *m*. The *jth* *IMF* component of the *ith* sequence is denoted by *IMFij*, and *Ri* denotes the residual term of the *ith* sequence.

### NAR dynamic neural network

The NAR dynamic neural network is based on the NAR nonlinear autoregressive model, which uses itself as a regressor variable and represents the random variables at a subsequent moment by a linear combination of random variables over time^32^. NAR, as a time series-based dynamic neural network, offers more than a mere static mapping in its output. It involves the comprehensive utilization of previous dynamic outcomes, enabling interconversion with feedforward networks. Hence, it possesses feedback and memory capabilities, demonstrating superior performance compared to feedforward neural networks^33^.

The NAR neural network model can be described as:

12 $$ y\left( t \right) \, = \, f\left( {y\left( {t \, - \, 1} \right), \, y\left( {t \, - \, 2} \right), \, \cdots \, , \, y\left( {t \, - \, d} \right)} \right) $$

where *y*(*t*) is the input value at the current moment, *y*(*t* *−* 1)*,* *y*(*t* *−* 2)*,…* *,* *y*(*t* *−* *d*) is the output value at the historical moment, and *d* is the delay order.

**Figure 1:**
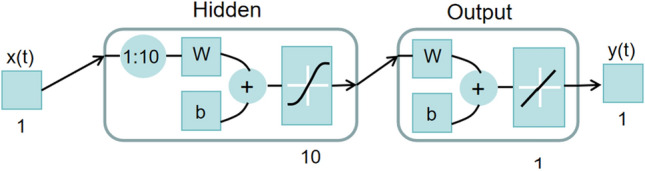
Schematic diagram of NAR dynamic neural network.

NAR dynamic neural networks generally consist of an input layer, a time lag layer, a hidden layer and an output layer. As shown in Fig. 1, the data *y*(*t*) is input from the input layer, processed, trained, and learned through the time lag and hidden layers, Finally, the output layer outputs the prediction results. where *y*(*t*) is the input data, *y*_0_(*t*) is the output data, 1*:*10 is the delay order, W is the connection weight, and *b* is the threshold value.

The NAR dynamic neural network model differs from other network models in two main aspects: Firstly, both the input and output values of this model are *y*(*t*). Secondly, it includes input delays within the hidden layer. The NAR dynamic neural network, in essence, is a static neural network with an input delay function where the order of delay determines the number of inputs for the neural network. Therefore, to enhance the accuracy of predictions using the NAR dynamic neural network, adjustments can be made by tuning the delay order, the number of nodes within the hidden layer, and the quantity of neurons.

The general steps for establishing a dynamic neural network model like NAR are outlined as follows:

*Step* 1: Apply the Cross-validation method to compute the bias and scaling factors for the decomposed wind speed subsequence data, determining the model's order.

*Step* 2: Partition the wind speed subsequence data into training, validation, and testing sets with specified proportions. Simultaneously, utilize MATLAB's 'preparets' function for data transformation.

*Step* 3: Define the number of neurons in the hidden layer and the delay order.

*Step* 4: Train the neural network model using in-sample data and assess the fitting effect through error autocorrelation plots. Repeat the process if the fitting criteria are not met.

*Step* 5: Preserve the trained neural network and proceed with data prediction, observing prediction errors.

Through the aforementioned steps, NAR dynamic neural network models were constructed for different wind speed subsequence data. Executing these models generated predictions for the wind speed subsequence data. However, the conventional approach for predicting wind speed sequences involves summing the predicted values of all components and trend terms to obtain the predicted wind speed sequence. This method overlooks the significant stochastic, uncertain, and nonlinear characteristics of wind speed data during prediction. This study introduces a mathematical analytical model to derive weights for different wind speed subsequence components. These weights are used to aggregate the predictions, enhancing the accuracy of the model's predictions.

The mathematical analytical model is mainly used to calculate the weight coefficients of each component by solving a specific mathematical model^34^, and the specific ideas and solution process are as follows:

The wind speed predictions by weight superposition are:

13 $$ \overline{x}\left( t \right) = c_{1} f_{1p} \left( t \right) + \cdots c_{n} f_{np} \left( t \right) + c_{n + 1} r_{np} \left( t \right) $$

where $c_{1} , \ldots ,c_{n} ,\;c_{n + 1}$ is the weighting factor corresponding to each component;$f_{1p} \left( t \right), \ldots f_{np} \left( t \right),r_{np} \left( t \right)$ is the prediction result of each component.

Here, the error sum of squares *P* is minimized as the objective function, i.e.

14 $$ P = \min \sum\limits_{t = 1}^{N} {\left[ {x\left( t \right) - \overline{x}\left( t \right)} \right]} $$

Substituting Eq. (10) into Eq. (11) yields:

15 $$ P = \min \sum\limits_{t = 1}^{N} {\left( {x\left( t \right) - c_{1} f_{1p} \left( t \right) - \cdots - c_{n} f_{np} \left( t \right) - c_{n + 1} r_{np} \left( t \right)} \right)}^{2} $$

The optimal weighting factor is found by finding the partial derivative of *P* with respect to each weighting factor such that it satisfies the following constraint.

16 $$ \left\{ \begin{gathered} \frac{\partial P}{{\partial c_{1} }} = - 2\sum\limits_{t = 1}^{N} {\left( {x\left( t \right) - c_{1} f_{1p} \left( t \right) - \cdots - c_{n} f_{np} \left( t \right) - c_{n + 1} r_{np} \left( t \right)} \right)c_{1p} (t) = 0} \hfill \\ \cdots \hfill \\ \frac{\partial P}{{\partial c_{n} }} = - 2\sum\limits_{t = 1}^{N} {\left( {x\left( t \right) - c_{1} f_{1p} \left( t \right) - \cdots - c_{n} f_{np} \left( t \right) - c_{n + 1} r_{np} \left( t \right)} \right)c_{np} (t) = 0} \hfill \\ \frac{\partial P}{{\partial r_{n + 1} }} = - 2\sum\limits_{t = 1}^{N} {\left( {x\left( t \right) - c_{1} f_{1p} \left( t \right) - \cdots - c_{n} f_{np} \left( t \right) - c_{n + 1} r_{np} \left( t \right)} \right)r_{np} (t) = 0} \hfill \\ \end{gathered} \right. $$

### Optimizing the HHT-NAR model prediction process

**Figure 2:**
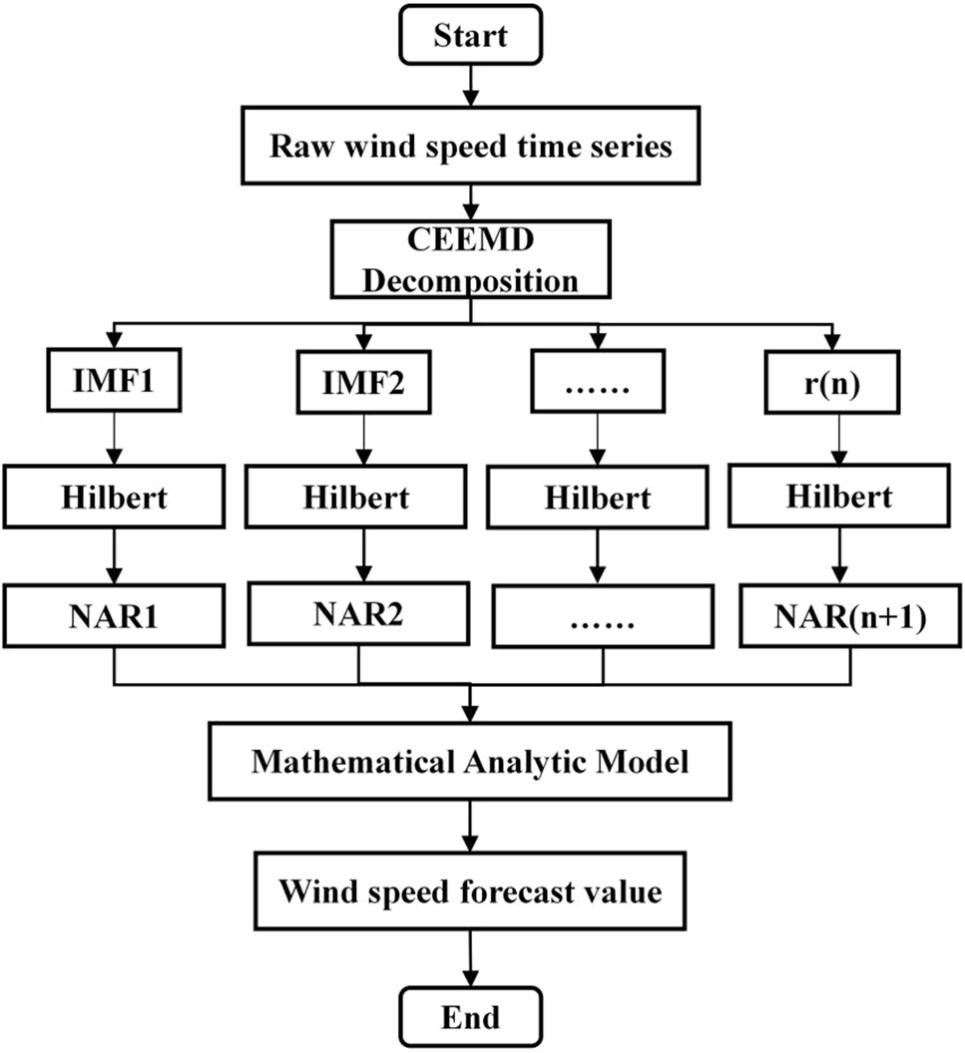
Flow chart of optimized HHT-NAR model.

Through the intricate process of optimizing the HHT-NAR model, we initiate by pre-processing the wind speed time series to ensure data quality and integrity. Subsequently, we employ the complementary ensemble empirical mode decomposition (CEEMD) for an efficient decomposition of the wind speed time series, acquiring Intrinsic Mode Functions (IMFs) of varying frequencies. These IMF components, processed through the Hilbert–Huang transform (HHT), effectively unveil the nonlinear and non-stationary features embedded within the wind speed signal. Employing mathematical analytical methods, we compute the weighting coefficients for each IMF to more accurately quantify the impact of CEEMD decomposition. Next, utilizing these weighted IMFs, we construct a dynamic Neural Autoregressive (NAR) model. Through training and optimization, the model is adapted to the historical variations in wind speed data. Ultimately, the refined model, post-optimization, can be effectively applied in practical scenarios, enabling precise short-term wind speed predictions. The detailed prediction process is depicted in Fig. 2.

The advanced techniques such as complementary ensemble empirical mode decomposition (CEEMD) and Hilbert–Huang transform (HHT) enable a more precise analysis of the nonlinear and non-stationary features within wind speed time series. These techniques enhance the adaptability of models to complex wind energy data, consequently boosting the accuracy of short-term wind speed predictions. Employing mathematical analytical methods to compute the weighting coefficients for Intrinsic Mode Functions (IMFs) quantifies the precision of CEEMD decomposition, further fortifying the model's robustness. This enhancement allows the model to better adapt to varying wind speed scenarios, thus improving its stability and reliability across different wind speed fluctuations^35,36^. Optimizing the HHT-NAR model workflow facilitates meeting the stability requirements for input signals in power systems, effectively addressing the challenges posed by the stochastic intermittency of natural winds on the power grid. This holds significant importance for the scheduling and control of wind farms, along with ensuring the secure and stable operation of power systems.

## Example of calculation and analysis of results

### Wind speed sequence experiment

Xinjiang Alashankou wind area is a wind resource rich area, the annual average wind power density in the center of the wind area is > 200 W/m^2^, and the effective wind speed hours are > 5500 h. And it has good wind resource conditions for large wind farms, and the wind speed in the area has no seasonal characteristics. The actual wind speed data of the region from 2021-6-15 to 2021-12-31 were selected for the arithmetic analysis, and the temporal resolution of the data was 1 day. After data processing, the first 80% of the data were taken as the experimental training set and the last 20% as the test set.

To assess the performance of the constructed prediction model, three error evaluation metrics were employed: Root Mean Square Error (RMSE), coefficient of determination (R^2^), and Mean Absolute Error (MAE) more accurately. RMSE and MAE serve as metrics to gauge prediction errors, where smaller values indicate closer proximity between predicted and actual values, signifying lower prediction errors. A higher R^2^ value nearing 1 indicates better fitting of the prediction model. The specific mathematical formulas for these metrics are provided as follows:

17 $$ RMSE = \sqrt {\frac{1}{N}\sum\limits_{i = 1}^{N} {\left( {y_{i} - \hat{y}_{i} } \right)^{2} } } $$

18 $$ MAE = \frac{1}{N}\sum\limits_{i = 1}^{N} {\left| {y_{i} - \hat{y}_{i} } \right|} $$

19 $$ R^{2} = SSR/SST = \sum\limits_{i = 1}^{N} {(\hat{y}_{i} - \overline{y}_{i} )}^{2} /\sum\limits_{i = 1}^{N} {(y_{i} - \overline{y}_{i} )}^{2} $$

In the formulas: *y*_*i*_ represents the actual value of wind speed at time *i*. $\hat{y}_{i}$ represents the predicted value of wind speed at time *i*. *N* denotes the total length of the wind speed sequence.

**Figure 3:**
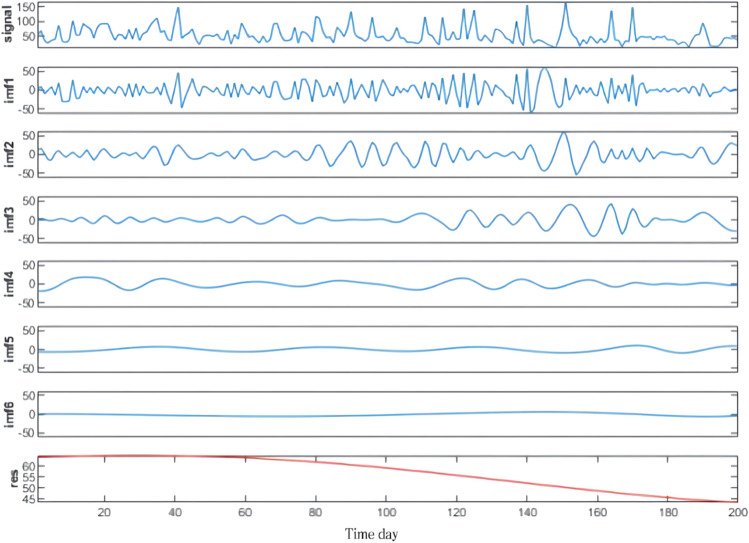
Wind speed CEEMD decomposition results.

**Figure 4:**
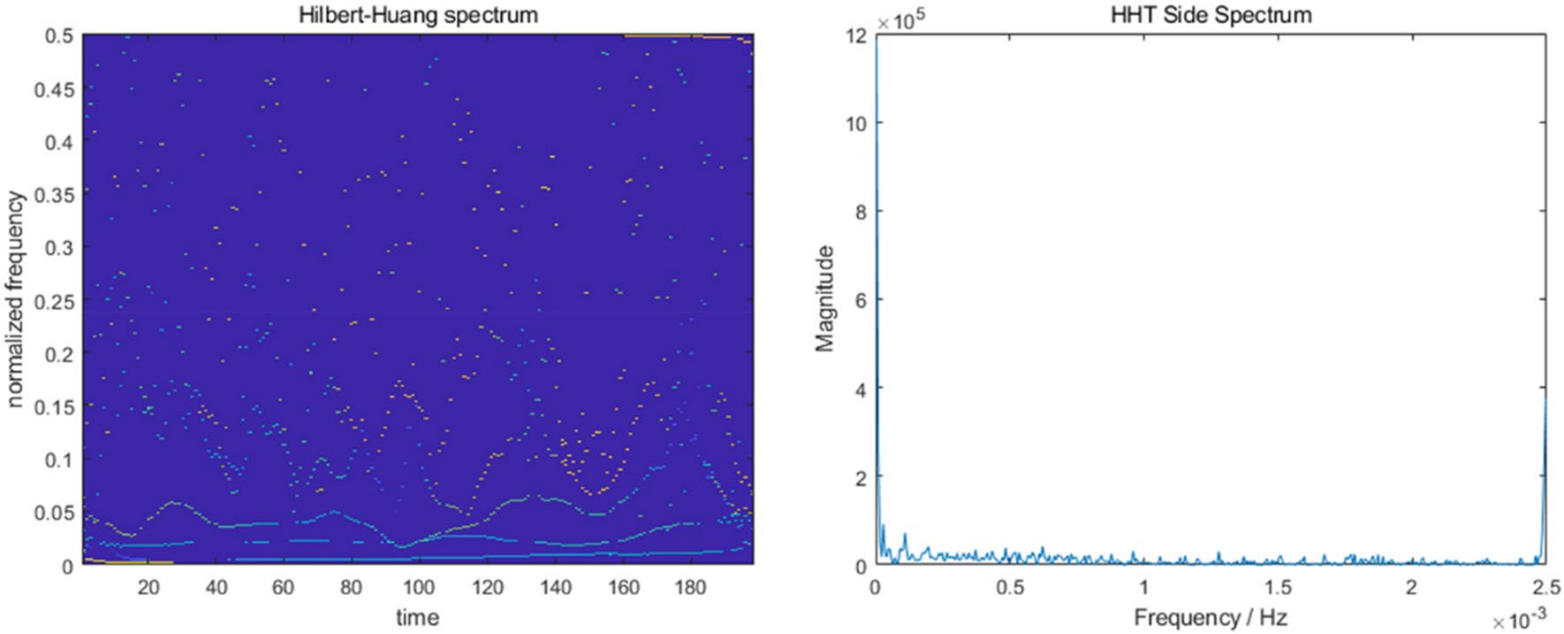
Wind speed HHT spectra and side spectra.

**Figure 5:**
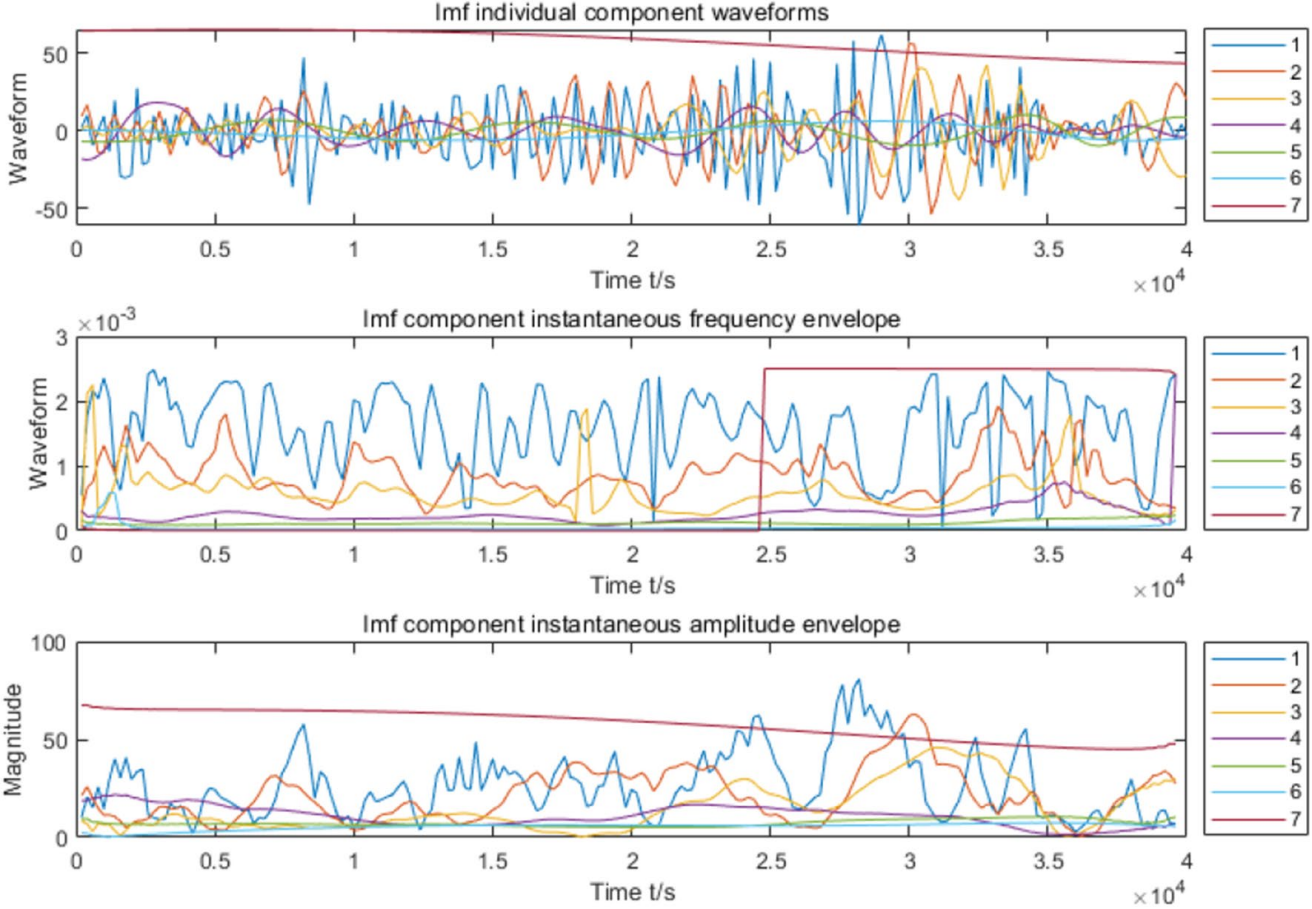
Waveform and amplitude of wind speed component.

The raw wind speed data and its CEEMD decomposition results are shown in Fig. 3, and a total of 6 IMF components and 1 residual component are decomposed. From Fig. 3, it is obvious that the temporal characteristic scale of the IMF component increases sequentially from IMF1 to IMF6, and its frequency changes from high to low. The spectrograms of wind speed and side spectrograms were then analyzed by HHT, as shown in Fig. 4, and the final waveforms of each component and frequency variation with time were obtained (Fig. 5).

The degree of correlation between IMF components and wind speed data was judged according to Pearson correlation coefficient for reconstructing the components, and the correlation results are shown in Table 1.

**Table 1:** Results of Pearson correlation coefficient analysis.

IMF Portion	IMF1	IMF2	IMF3	IMF4	IMF5	IMF6	Res
Pearson correlation coefficient	0.5391	0.4914	0.3620	0.2315	0.1442	0.0119	0.2097

From the data presented in Table 1, it is evident that the wind speed components IMF1, IMF2, IMF3, IMF4, and the trend term exhibit relatively high correlation coefficients, indicating a stronger association with the original data. However, IMF5 and IMF6 display weaker correlations with the original data. The component reconstruction method with minimum error and simpler operation is used, and the components with large correlation degree are substituted into Input1 ∼ Input 5 components and input to the NAR neural network for prediction, while the IMF5 and IMF6 with smaller correlation degree are summed and reconstructed into a new component Input 6 input to the neural network, and the difference in order of magnitude between IMF5 and IMF6 is not significant, and data normalization is not required.

The number of neurons in the hidden layer of each input is first referred to the literature for value selection reference^37^. The result obtained from the empirical formula is used as the initial value, and then the experimental method is used to continuously adjust the value to select the most suitable value. After repeated experimental comparison and analysis, the settings of the hidden layer neurons corresponding to different components in this experiment are shown in Table 2.

**Table 2:** Number of neurons in the hidden layer corresponding to different components.

Input component	Input1	Input2	Input3	Input4	Input5	Input6
Number of implied neurons	8	10	10	12	12	12

**Figure 6:**
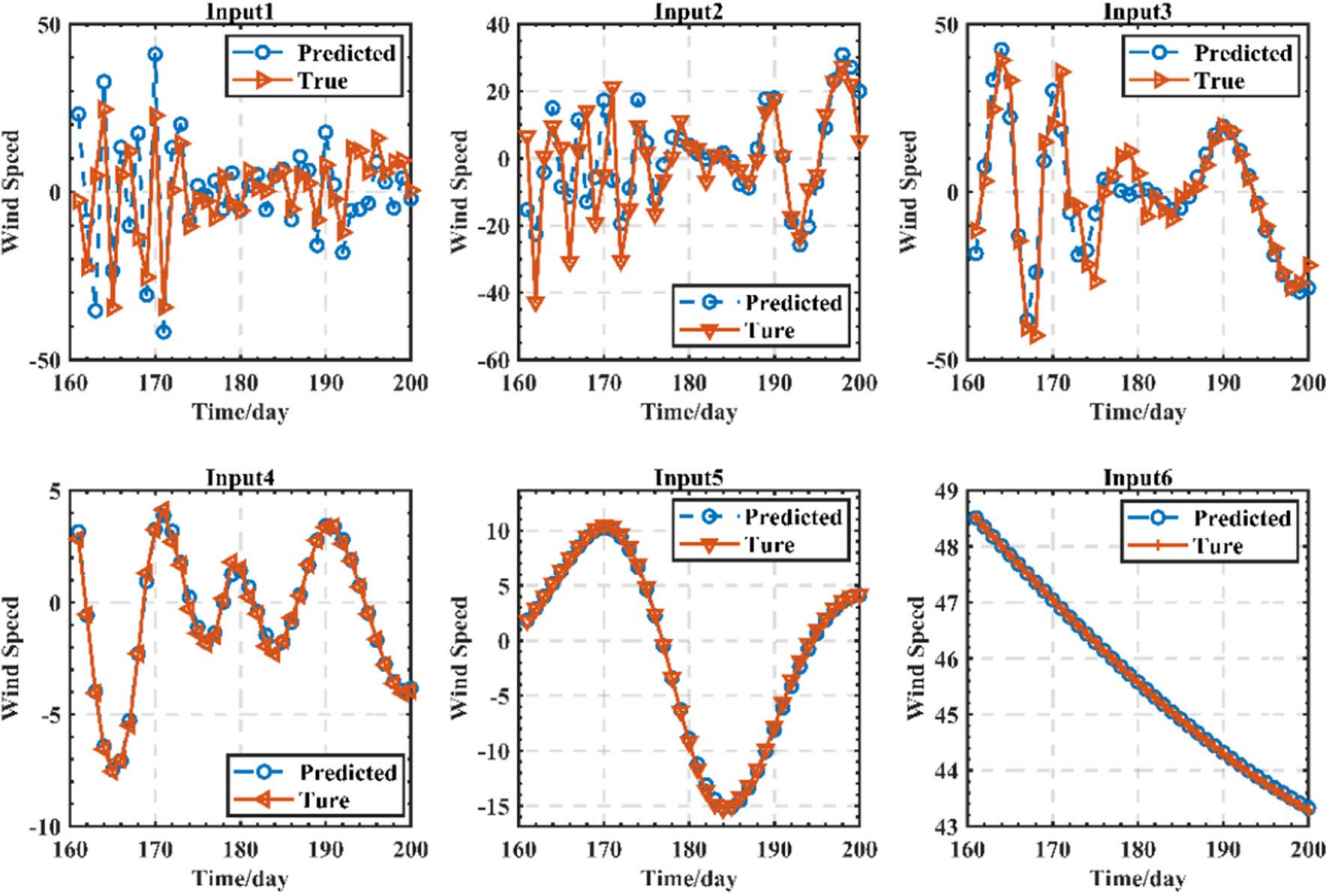
Input prediction of each component.

The optimal NAR dynamic neural network model for each waveform is established to improve the prediction accuracy, and it can be seen from Fig. 6 that the smoothness of the wind speed time series is improved, and the volatility is significantly reduced after CEEMD decomposition. The prediction errors from Input 1–Input 6 are getting lower and lower, which indicates that the training effect is gradually getting better.

**Figure 7:**
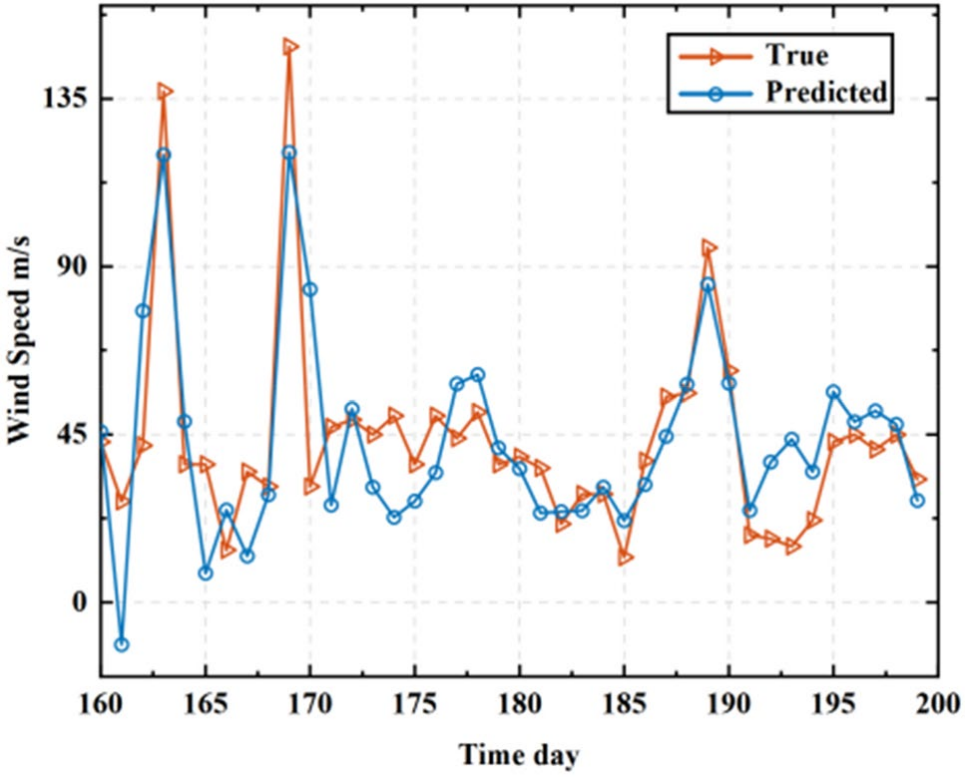
Comparison between the optimized model's predicted values and the original wind speed sequence.

The predicted values of Input 1 to Input 6 were summed to obtain the predicted values of the original wind speed sequence, as depicted in Fig. 7, illustrating their comparison with the actual values.

The computation of three error assessment metrics is shown in Table 3: the optimized model exhibits an RMSE of 18.9818 m/s, an MAE of 14.1063 m/s, and an R^2^ value of 0.8827. Smaller values of RMSE and MAE indicate a closer proximity between predicted and actual values, signifying smaller prediction errors. Meanwhile, an R^2^ value closer to 1 suggests a better fitting of the prediction model. Thus, the model established in this article is deemed suitable for forecasting actual wind speeds within the studied region.

**Table 3:** The error assessment results for the optimized model.

Model	Error indicators		
	RMSE (m/s)	R^2^	MAE (m/s)
Optimization of HHT-NAR	9.9818	0.8927	7.1063

### Day-level wind speed prediction based on optimized HHT-NAR model

**Figure 8:**
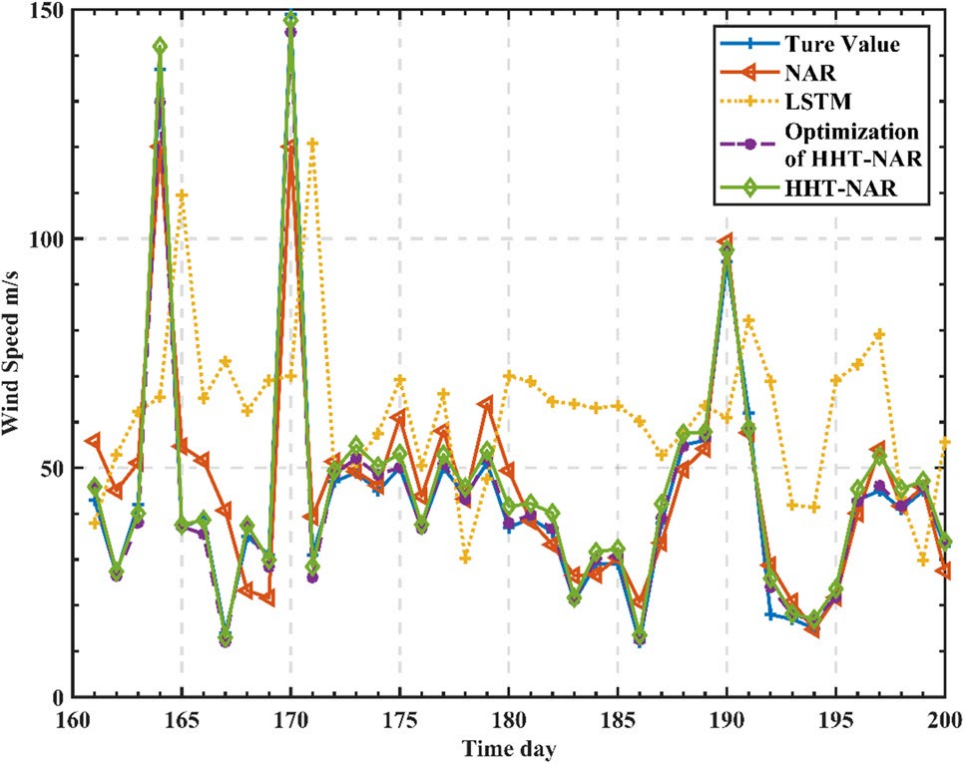
Comparison of actual wind speed at Alashankou station with predictions from various models.

In order to better validate the effectiveness of the proposed model, the article selects the long short-term memory neural network (LSTM) model that introduces self-loop on top of RNN, which is capable of capturing long-range dependency and nonlinear information^38–40^. A single LSTM and NAR prediction model was developed for the raw wind speed data, and a comparison of the prediction results of several models is shown in Fig. 8.

As seen in Fig. 8, the single LSTM prediction is poor, with a certain time lag, and it is often difficult to track the prediction when the wind speed varies widely. The single NAR prediction is slightly better than LSTM, and there is no lag in the abrupt response to wind speed data, but it cannot predict individual points more accurately. The overall prediction effect of the HHT-based NAR dynamic neural network prediction model is improved, and there is basically no lag, but there is also a large error in individual mutation points, and the optimized HHT-NAR model reduces the error value in wind speed. The results showed that the optimized wind speed model of HHT-NAR reduced the prediction error compared with other wind speed-based prediction methods and obtained higher prediction accuracy than other deep neural architectures. The error evaluation results of each model are shown in Table 4.

**Table 4:** The error assessment results for different prediction models.

Error indicators	Models			
	LSTM	NAR	HHT-NAR	Optimization of HHT-NAR
RMSE (m/s)	37.0197	10.3966	5.9786	2.5375
R^2^	0.7941	0.8585	0.8915	0.9215
MAE (m/s)	29.5313	7.6396	2.725	1.8072

From Table 4, it is evident that the optimized HHT-NAR model constructed in this paper performs the best in predicting results, with RMSE, R^2^, and MAE values of 2.5375 m/s, 0.9215, and 1.8072 m/s, respectively. In comparison, the HHT-NAR dynamic neural network has RMSE, R^2^, and MAE values of 5.9785 m/s, 0.8915, and 2.725 m/s, respectively. When comparing the optimized HHT-NAR model to the HHT-NAR model, there is a decrease of 57.56% and 33.68% in RMSE and MAE, respectively, while R^2^ has increased by 3.37%. The optimized HHT-NAR model demonstrates a significant reduction in RMSE and MAE, indicating closer proximity of predicted values to actual values, thereby minimizing prediction errors and further improving prediction accuracy. Comparing the NAR dynamic neural network model to the LSTM model, there is a reduction of 71.9% and 27.6% in RMSE and MAE, respectively, with an increase of 7.5% in R^2^. Furthermore, comparing the HHT-NAR dynamic neural network combined model to the NAR dynamic neural network model, there's a decrease of 75.59% and 31.10% in RMSE and MAE, respectively, with a 7.3% increase in R^2^. This demonstrates that optimizing the HHT-NAR model for decomposing highly nonlinear and random original wind speed data significantly enhances prediction accuracy, showcasing promising feasibility.

### Annual wind speed forecast at Karamay station

**Figure 9:**
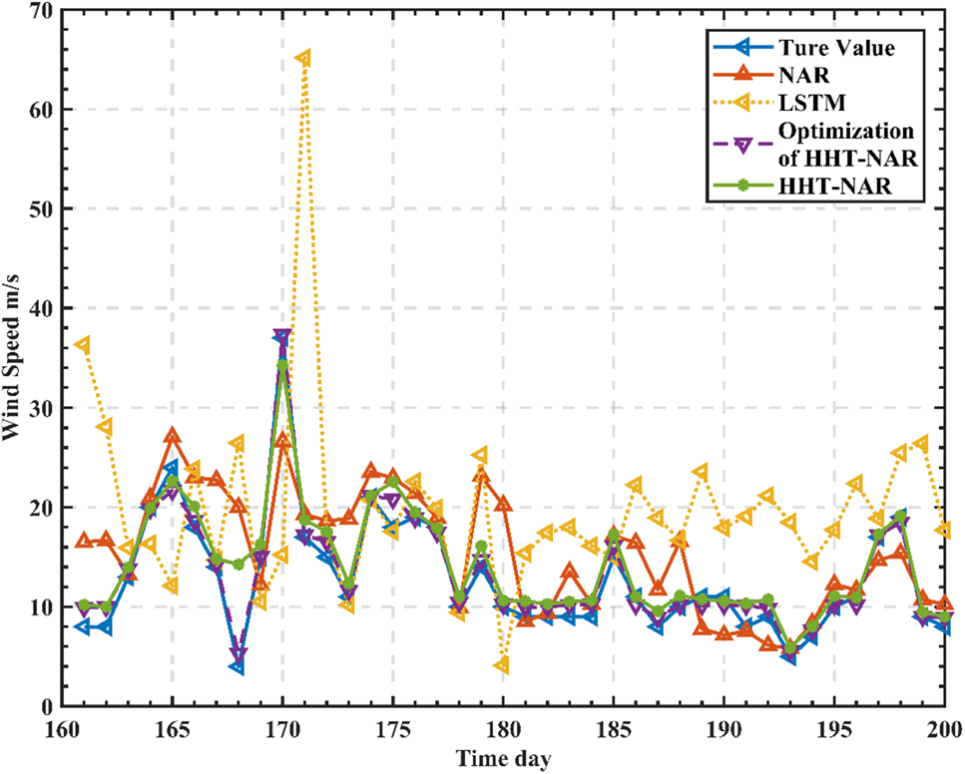
Comparison of actual wind speed at Karamay station and prediction of each model.

In order to further verify the universality and accuracy of the model proposed in this paper, the prediction object in this example is not limited to the wind energy rich area, but also made prediction for the wind energy sub-rich area in Karamay, Xinjiang, using the wind speed data of Karamay wind farm for the years 2021-6-15–2021-12-31 to do short-term wind speed prediction test. A total of 200 data points were selected, and the last 40 data points were selected as the test set. Figure 9 illustrates the comparison between the actual wind speed at the Karamay station and the predictions from various models.

From Table 5, it can be observed that the predictive errors of the three models in the wind energy sub-enrichment area are smaller compared to the previous station, attributed to lower fluctuations in wind speed sequences in this region. Notably, the optimized HHT-NAR dynamic neural network model proposed in this paper outperforms other comparative models significantly, demonstrating a pronounced effect in improving the root mean square error (RMSE) over the HHT-NAR model. This validates the universality and accuracy of the method proposed in this study. Furthermore, the optimized HHT-NAR dynamic neural network model shows a 5% to 47% enhancement in the coefficient of determination (R^2^) compared to the other three models, indicating its superior predictive performance.

**Table 5:** Error evaluation results of different prediction models.

Error indicators	Models			
	LSTM	NAR	HHT-NAR	Optimization of HHT-NAR
RMSE (m/s)	13.1963	8.3574	5.2322	1.9977
R^2^	0.6132	0.7521	0.8637	0.9037
MAE (m/s)	9.3827	3.9614	1.4799	0.8254

### The analysis and discussion of forecast results

From Figs. 8 and 9, it is evident that all four models generally depict the overall trend of wind speed data over time. However, in comparison to the actual measurements, the standalone LSTM model inadequately captures the finer details of the data. Specifically, it exhibits significant prediction errors for days with higher wind speeds, attributed to the strong random nature of the wind speed sequence that interferes with the model's predictive accuracy.

Comparatively, the predictions made by the NAR model are closer to the actual measurements for days with higher wind speeds and present more detailed data for other days. This is due to the NAR model's better handling of issues related to gradient vanishing and explosion during long sequence training, resulting in more comprehensive performance.

Additionally, the predictive performance of the HHT-NAR model surpasses that of the NAR model. It demonstrates closer alignment with the actual measurements on days with higher wind speeds, indicating that decomposing the wind speed sequence enhances the model's accuracy. The optimized HHT-NAR model exhibits the best predictive performance, accurately forecasting even on days with higher wind speeds. This improvement is attributed to CEEMD, which effectively isolates different fluctuation characteristics within the precipitation sequence. Furthermore, CEEMD decomposes the added noise, reducing reconstruction errors. Using mathematical analysis to predict the decomposed components based on different weights allows the model to capture the changing characteristics of each component more effectively, significantly enhancing prediction accuracy.

## Conclusion

1. Facing the intermittence and volatility of wind speed time series, this study utilized the CEEMD algorithm to decompose the wind speed sequence. Combined with the HHT method, this approach unearthed the physical characteristics of wind speed, facilitating the construction of a NAR dynamic neural network model suitable for prediction purposes.
2. The application of the CEEMD algorithm effectively reduced the non-stationarity of the original sequence, while the HHT method adeptly uncovered the nonlinear and non-stationary characteristics of the wind speed signal, laying a robust foundation for constructing the NAR dynamic neural network model. Utilizing mathematical analysis to examine the weight coefficients of each component, we successfully quantified the impact of the insufficient precision in the CEEMD decomposition. It is recommended for future work to further optimize the HHT-NAR model, especially in the selection of mathematical analysis models, employing more refined methods to enhance the model's fitting capability.
3. The optimized HHT-NAR dynamic neural network model constructed in this study has achieved significant success in wind speed prediction at two sites in Xinjiang. The model demonstrated exceptional performance in fitting the majority of wind speed data transition points, reducing RMSE and MAE in both wind-rich and wind-limited areas, exhibiting excellent fitting accuracy. For future considerations, efforts can be directed towards refining the combined model to converge to optimal precision while maintaining higher predictive stability.
4. In further optimizing the HHT-NAR model, the paper acknowledges the need for more in-depth comparative studies. Future work will extend the comparison beyond the machine learning models mentioned in this study. Additionally, exploration into more complex algorithms and models, such as optimizing combined prediction strategies for enhanced precision and stability, is proposed. This endeavor aims to further enhance the accuracy and stability of wind speed prediction, offering a more reliable forecasting tool for wind energy generation. This endeavor aligns with contributing more significantly to the sustainable development of renewable energy sources.

## Data Availability

Datasets and other materials are available with the authors and may be accessible at any time upon request.
